# The effect of birth preparedness and complication readiness on skilled care use: a prospective follow-up study in Southwest Ethiopia

**DOI:** 10.1186/1742-4755-11-60

**Published:** 2014-08-05

**Authors:** Gurmesa Tura, Mesganaw Fantahun Afework, Alemayehu Worku Yalew

**Affiliations:** 1 Department of Population and Family Health, College of Public Health and Medical sciences, Jimma University, Jimma, Ethiopia; 2 Department of Reproductive Health and Health Service Management, School of Public Health, Addis Ababa University, Addis Ababa, Ethiopia; 3 Department of Epidemiology and Biostatistics, School of Public Health, Addis Ababa University, Addis Ababa, Ethiopia

**Keywords:** Birth preparedness, Complication readiness, Skilled care, Southwest Ethiopia

## Abstract

**Abstracts:**

**Background**

Skilled care during and immediately after delivery has been identified as one of the key strategies in reducing maternal mortality. However, recent estimates show that the status of skilled care during delivery remained very low in Ethiopia. Birth preparedness and complication readiness has been implemented as comprehensive strategy to fill this gap. However, its effectiveness in improving skilled care use hasn’t been well studied.

**Objective**

The objective of this study was to determine the effect of birth preparedness and complication readiness on skilled care use in Southwest Ethiopia.

**Methods**

A prospective follow-up study was conducted from September 2012-April 2013 in Southwest Ethiopia among randomly selected 3472 mothers. Data were collected by using pre-tested interviewer administered questionnaires and analyzed by using SPSS for windows V.20.0 and STATA 13. Mixed-effects multilevel logistic regression model was used to look at the relation between birth preparedness and complication readiness plan and skilled care use and identify other determinant factors.

**Results**

The status of skilled care use was 17.5% (95% CI: 16.2%, 18.8%). Factors affecting skilled care use existed both at the community as well as individual levels. Planning to use skilled care during pregnancy was found to increase actual use significantly (OR = 2.24; 95%CI: 1.60, 3.15). Place of residence, access to basic emergency obstetric care, maternal education, husband’s occupation, wealth quintiles, number of pregnancy, inter-birth interval, knowledge of key danger signs during labor and ANC use were identified as factors affecting skilled care use.

**Conclusions**

The status of skilled care use was found to be low in the study area. Birth preparedness and complication readiness had significant effect on skilled care use. Socio-demographic, economic, access to health facility, maternal obstetric factors and antenatal care were identified as determinant factors for skilled care use. Designing appropriate interventions to improve information, education and communication, antenatal care use, family planning and knowledge of key danger signs are recommended.

## Introduction

The fifth Millennium Development Goal (MDG_5_) calls for the reduction of Maternal Mortality Ratio (MMR) by 75% between 1990 and 2015. However, only 47% decline had been achieved till 2011 globally [1]. As a result, about 287,000 mothers die because of problems related to pregnancy and child birth each year. About 99% of this occurs in developing countries. Sub-Saharan Africa and Southern Asia account for 85% [2]. This high maternal mortality in developing countries has been largely attributed to the low coverage of skilled care use during delivery [3]. Skilled care during and immediately after delivery and emergency obstetric care have also been identified as key strategies and one of the indicators to track the MDG in reducing maternal mortality. However, recent estimates show that the proportion of deliveries attended by skilled attendant in many African countries remained below 50% [4,5].

Ethiopia is also among the countries committed to achieve the MDG_5_ target by reducing maternal mortality by three-quarter. However, the decline in the last 15 years was found to be non-significant with MMR of 676 per 100,000 live-births in the Ethiopian Demographic and Health Survey (EDHS) of 2011 as compared to 673 and 871 per 100,000 live-births in EDHS 2005 and EDHS 2000 respectively. This in turn is also explained by the low coverage of skilled care during delivery in the country. The proportion of births attended by skilled attendant in the country had long been below 5% and hardly reached 10% in 2011 [6-8].

Birth preparedness and complication readiness (BP & CR) has been suggested by the World Health Organization (WHO) as a comprehensive approach for increasing coverage of skilled delivery care and reducing the three delays to care seeking during obstetric emergencies. Many countries in Sub-Saharan Africa including Ethiopia have adopted this approach and included in the routine focused antenatal care. However, its effectiveness has not been well studied [9,10]. Even, the existing studies show conflicting and non-conclusive findings. In a cross-sectional study conducted in India, BP & CR had significant effect on skilled care use [11]. In the contrary, in a field trial study conducted in Nepal, it had non-significant effect [12].

Therefore, for policy and program improvement, it is important to have up-to-date information on the status of BP & CR and its actual effect on skilled care use in different local contexts. Moreover, it is crucial to study whether intention really implies action in skilled care use during delivery. However, though we have few cross-sectional studies on the status, studies on the effectiveness of this approach in increasing skilled care during delivery by strong analytic designs are limited in Africa and non-existent in Ethiopia.

Thus, the purpose of this study was to fill this gap by determining the effect of BP & CR on skilled care use by conducting community based prospective follow-up study in Southwest Ethiopia. The findings of the study will also be used by policy makers and program implementers so as to improve the coverage of skilled care during delivery and achieve the stated MDG target in reducing maternal mortality.

## Methods

### Study design and setting

This community based prospective follow-up study was conducted in Jimma Zone, located 346kms Southwest of Addis Ababa, the capital city of Ethiopia, from September 2012-April 2013. Jimma Zone is one of the 17 Zones of the Oromia Regional State of Ethiopia, which was named for the former Kingdom of Jimma and absorbed into the former province of Kaffa in 1932. The Zone has a total of 17 rural districts called ‘Woredas’ and two town administrations with an estimated total population of about 2.6 million and a male-to-female ratio of 1.01:1. The great majority (89%) of the population of the Zone were rural residents. The Zone has a total of 521,506 households with an average household size of 4.77 persons per a household. The potential health service coverage of the zone for the year 2011 was about 52% [13,14].

### Source population and study participants

The source or target population considered for this study was all women who had given birth during the study period, from September 2012-April 2013 in Jimma Zone. However, as this was a prospective follow-up study, the study participants were all pregnant women identified based on the sampling procedure and enrolled in the study at a base line and had been followed till 28 days post-partum period. At the baseline, the status of BP & CR and affecting factors were studied which is under review for publication on Pan African Medical Journal. This study was the intermediate one collected during the second phase just at the end of neonatal period. The final study, determinants and causes of neonatal mortality, is again on peer review process for publication on Plos One.

### Sample size and sampling technique

The minimum required sample size for this study was determined by using Epi-Info V.3.5.1. by considering two sample comparisons of proportions based on the following assumptions. The outcome variable was skilled care use and the explanatory variable was BP & CR plan. As there was no similar study conducted in the country to be used as a base to determine the sample size, study from other developing country was used. In a study done in India, BP & CR was found to increase the skilled care use by 80% [11].

In Ethiopia, the proportion of women attended by skilled delivery attendant was 10% and this was taken as the proportion among non-exposed group (P_1_ = 0.1) [8]. The proportion of women attended by skilled attendant among exposed (prepared for birth and its complication) was estimated to be 18% (P_2_ = 0.18) to detect 8% difference or 80% increment. A level of confidence of 95% and power of 90% were considered. In addition, the coverage of exposure from the general population was estimated to be 22% [15]. Thus, a ratio of 1:4 (r = 4) was used for exposed-to-non-exposed. A design effect of 2 was also considered because of the multistage clustered sampling techniques. Finally, 10% was added for non responses and miss to follow-up and the final sample size became 2603 mothers. However, this study was part of a big longitudinal study in which 3612 pregnant women had been on follow-up to look at the determinants of neonatal mortality. As a result, after excluding lost-to-follow-up and abortion cases, 3472 mothers were included in the analysis of this study.

Multi-stage clustered sampling technique was used to identify pregnant women for the study. Initially, the Zone was stratified as Town Administrations (2 in number) and rural districts (17 in number). Then, at first stage, 5 districts were selected randomly from the 17 rural districts (‘Woredas’). At second stage, all the selected 5 districts were stratified in to urban and rural ‘Kebeles’ (A ‘kebele’ is the smallest administrative unit having 5000 population in average and considered as clusters in this study). Then, by simple random sampling method, 9 rural ‘Kebeles’ and 2 urban ‘Kebeles’ were selected from each selected district. Jimma town administration and Agaro town administration have 13 and 5 ‘Kebeles’ respectively and all were included purposefully. Then, for all the selected ‘kebeles’, pregnant women were enumerated by using house-to-house visit and all obtained and registered were included in the study. All women who reported to have pregnancy of 12 weeks or above as defined by loss of three consecutive menses were considered as eligible and enrolled in the study.

### Data collection process

Pre-tested interviewer administered structured questionnaire was adapted from the safe motherhood questionnaire developed by maternal and neonatal health program of JHPIEGO to measure the composite variable for birth preparedness and complication readiness [9]. The indicators for the wealth index were adapted from EDHS [8]. To control the quality of data, training, pretest, supervision and use of local languages were made.

### Data management and analysis

The collected data were coded and entered into EpiData V.3.1 to minimize logical errors and design skipping patterns. Then, the data were exported to SPSS for windows version 20.0 for cleaning, editing and analysis. Descriptive analysis was made by computing proportions and summary statistics. Socio-economic quintiles were determined by using Principal Component Analysis (PCA). As Jimma and Agaro town administrations were both purposefully included, the status of skilled care use was estimated by calculating weighted percentage based on the complex sample survey procedure.

Bivariate analysis was done by using cross-tabulation to see associations between each independent variable and skilled care use. All variables having P < 0.25 were considered as candidates for the final model. As multistage clustered sampling method was used because of the different levels of factors, mixed-effects multilevel logistic regression model was used by using STATA 13 to identify factors having significant association with skilled care use. ‘Kebeles’ were considered as clusters and kebele level variables such as place of residence and access to basic emergency obstetric care (BEmOC) and comprehensive emergency obstetric care (CEmOC) were taken as higher level (level-2). Mothers were nested within their households and the community. As a result, maternal individual variables including socio-demographic, economic, obstetric and BP & CR were taken as lower level (level-1). Goodness of fit of the multilevel model was tested by the log likelihood ratio (LR) test. Multicollinearity between the independent variables was assessed by using variance inflation factor (VIF). As all included variables had VIF < 10, no multicollinearity was detected. In addition cross-level two-way interactions were checked; but, no significant interaction was detected.

### Ethical clearance

Ethical approval was obtained from the Institutional Review Board (IRB) of College of Health Sciences of Addis Ababa University. Necessary permission was secured from all local administrators. Written informed consent was obtained from each respondent before actual data collection. Issues of confidentiality were maintained by removing any identifiers from the questionnaire. To protect vulnerable group, data collectors were trained to maintain confidentiality and provide necessary health information based on the need of the participants, but not an intervention.

### Operational definition

#### Birth preparedness and complication readiness

A package of interventions measured as a composite variable of 5 items: planed to save money, planed to arrange transport, planed to give birth in health facility, planed to be attended by skilled attendant and planed to arrange blood donor. Mothers who fulfilled three or more of the five items were considered as ‘well prepared’ and otherwise ‘not well prepared’.

#### Identified blood donor

The blood donation system in Ethiopia is based on both volunteer donors and replacement based. In this study, women who had identified volunteer family member to donate the blood during delivery if needed were considered as arranged blood donor and otherwise not.

#### Skilled care

Delivery attended in health facilities (hospital, health center or private clinics) attended by skilled attendants (doctor, midwife, nurse, health officer or unspecified health worker who has a training of Diploma or above). Delivery attended at health post by HEW was not considered as skilled care.

## Results

### Socio-demographic characteristics

A total of 3472 respondents were included in the analysis for this study. Majority, 2618 (75.4%), were rural residents. Most, 2227 (64.2%), were in the age group of 20-29 years with the mean age of 26.5 ± 5.0. Oromo was the dominant ethnic group, 3041 (87.6%) and Muslim was the leading religion, 3032 (87.3%). The great majority, 3289 (94.8%), were housewives by occupation and more than half, 1878(54.1%), didn’t attend any formal education (Table 1).

**Table 1:** Socio-demographic characteristics of respondents, Jimma Zone, Southwest Ethiopia, September 2012-April 2013 (n = 3472)

**Variables**	**No.**	**%**
**Residence**		
Urban	854	24.6
Rural	2618	75.4
**Age (Years)**		
15-19	174	5.0
20-24	974	28.1
25-29	1253	36.1
30-34	739	21.3
35-39	302	8.6
40-44	30	0.9
**Ethnicity**		
Oromo	3041	87.6
Amhara	169	4.9
Dawuro	97	2.8
Others*	165	4.7
**Religion**		
Musilim	3032	87.3
Orthodox	342	9.9
Protestant	98	2.8
**Marital Status**		
In marital union	3452	99.4
Not in marital union^†^	20	0.6
**Educational status**		
No formal education	1878	54.1
1-4	811	23.4
5-8	462	13.3
9-12	255	7.3
>12	66	1.9
**Occupation**		
Housewife	3289	94.8
Employed (Gov’t, NGO & Private)	81	2.3
Others‡	102	2.9
**Husband’s Occupation**		
Farmer	2473	71.2
Employed (Gov’t, NGO & Private)	373	10.7
Merchant	412	11.9
Others^‡^	214	6.2

*Yem, Kaficho, Guraghe & Tigrie, † Single, divorced & widowed, ^‡^Merchant, student, daily laborer.

### Maternal health care use during pregnancy and delivery

Of all the 3472 respondents, 2634 (75.9%) had at least one ANC visit during pregnancy and 1830 (52.7%) had used either from hospital or health center attended by skilled attendant. However, only 1228 (35.4%) had four or more visits and 572 (16.5%) started during first trimester. Of the respondents, 1064 (30.6%) used skilled care during delivery (74.1% in urban and 16.5% in rural). However, the weighted coverage of skilled care use was much lower 17.5% (95% CI: 16.2%, 18.8%). The major reasons for giving birth in hospital or health center were pre-planed (43.5%), need skilled attendant (41.0%), problem during labor (28.9%) and need clean/safe place (15.1%). Whereas, the most common reason for home delivery was lack of transport (31.1%) followed by home delivery is the usual place (24.0%) and more comfortable (23.1%) (Table 2).

**Table 2:** Skilled care use during pregnancy and delivery among respondents, Jimma Zone, Southwest Ethiopia, September 2012-April 2013 (n = 3472)

**Variables**	**No.**	**Unweighted %**	**Weighted %**
**ANC (at least once)**			
Yes	2634	75.9	72.2
No	838	24.1	27.8
**Place of ANC (n = 2634)**			
Hospital	145	5.5	1.2
Health Centre	1585	60.2	55.5
Private clinic/FGAE	100	3.8	0.7
Health Post	804	30.5	42.6
**Attendant of ANC (n = 2634)**			
Skilled attendant^§^	1830	69.5	57.4
HEW	804	30.5	42.6
**# of ANC visit (n = 2634)**			
1-3	1406	53.4	61.7
≥4	1228	46.6	38.3
**GA at ANC start (n = 2634)**			
1st trimester	572	21.7	16.3
2nd trimester	1771	67.2	72.0
3rd trimester	291	11.0	11.7
**Place of delivery**			
Hospital	425	12.2	6.4
Health Centre	573	16.5	10.7
Private clinic/FGAE	66	1.9	0.4
Health Post	48	1.4	1.8
Home/TBA’s Home	2360	68.0	80.7
**Delivery attendant**			
Skilled attendants^§^	1064	30.6	17.5
HEW	59	1.7	2.2
Family/TBA	2349	67.7	80.3
**Mode of delivery**			
SVD	3336	96.1	96.8
C/S	80	2.3	1.5
Assisted	56	1.6	1.7
**Reasons for health facility delivery (n = 1112, multiple response)**			
Pre-planned	484	43.5	
Need skilled attendant	456	41.0	
Problem during labor	321	28.9	
Need clean/safe place	168	15.1	
**Reasons for home delivery (n = 2360, multiple response)**			
Lack of transport	735	31.1	
Home delivery is my usual place	567	24.0	
Home delivery is more comfortable	546	23.1	
Lack of money for transport	289	12.2	
Lack of money for service cost	142	6.0	

^§^Doctor, nurse, midwife, HO, unspecified health workers.

### Intention vs action in skilled care use

A total of 1858 (53.5%) had planned to have 4 or more ANC visits from skilled attendants. However, only 1228 (35.4%) actually had 4 or more ANC visits. Using 4 or more ANC visit was 46.0% among those who planned to have it as compared to 23.2% among who didn’t plan which is statistically significant (COR = 2.82; 95%CI: 2.43, 3.27). Similarly, 1136 (32.7%) had planned to use skilled care during labor and 1064 (30.6%) actually used it. About 61.7% of those who had already planed actually used as compared to only 15.5% of who didn’t plan which is statically significant (COR = 8.76; 95% CI: 7.44, 10.32).

Four hundred thirty five (38.3%) of those who had already planned to use skilled care didn’t use it actually. The main reasons were the labor was not associated with problems 382 (87.8%), lack of transport 64 (14.7%) and lack of money for transport and services 58 (13.3%). On the other hand, 363 (15.5%) of those who didn’t plan to use skilled care actually used it. The main reason was occurrence of unanticipated problems at labor 174 (47.9%) among which prolonged labor was the leading one 152(41.9%). The other reason was family insisted at labor to have safe and clean delivery 99 (27.3%). This indicated that intention significantly implies action in skilled care use; however, not 100% as complication during labor is unpredictable (Table 3).

**Table 3:** Intention vs action in skilled care use during pregnancy and child birth and reasons among respondents, Jimma Zone, Southwest Ethiopia, September 2012-April 2013 (n = 3472)

**Plan (intention)**	**Actual practice (Action)**			**P-value**
	**Had ≥ 4 ANC Visit**			
**Planed ≥ 4 ANC Visit**	**Yes n(%)**	**No n(%)**	**Total n(%)**	P < 0.001 (COR = 2.82; 95%: 2.43, 3.27)
Yes	854 (46.0)	1004 (54.0)	1858 (53.5)	
No	374 (23.2)	1240 (76.8)	1614 (46.5)	
Total	1228 (35.4)	2244 (64.6)	3472 (100.0)	
**Planned to use skilled care during delivery**	**Used skilled care during delivery**			P < 0.001 (COR = 8.76; 95%: 7.44, 10.32)
	**Yes n(%)**	**No n(%)**	**Total n(%)**	
Yes	701 (61.7)	435 (38.3)	1136 (32.7)	
No	363 (15.5)	1973 (84.5)	2336 (67.3)	
Total	1064 (30.6)	2408 (69.4)	3472 (100.0)	
**Reasons for not using skilled care once planed (n = 435)**			**No.**	**%**
Labor not associated with problems (short and comfortable labor)			382	87.8
Lack of transport			64	14.7
Lack of money			58	13.3
**Reasons for using skilled care which was not planed (n = 363)**				
Any problem encountered during labor			174	47.9
Prolonged labor (>12 hours)			152	41.9
Family insisted at labor to have safe and clean delivery			99	27.3

### Factors affecting skilled care use

To evaluate the applicability of the mixed-effects multilevel logistic regression model, the Intra-class Correlation Coefficient (ICC (*ρ*)) was calculated in the empty model and it was found to be 0.489 indicating that 48.9% of the variation was contributed by between cluster variation. The test of the preference of log likelihood Vs logistic regression was also strongly significant (P < 0.001). Then, the full model was run by including all the cluster level and individual level variables and the ICC (*ρ*) became 0.113 indicating that 11.3% of the variation was attributed to cluster level variables again suggesting the preference of multilevel analysis. The preference of log likelihood Vs logistic regression was still strongly significant (P < 0.0001).

After adjusting in the final two-level mixed-effects logistic regression model, both the cluster level variables and individual level variables were found to be important determinants of skilled care use. Among the higher (cluster) level variables, place of residence and access to BEmOC were found to have statistically significant association with skilled care use. Women in urban areas were more than two times more likely to use skilled care as compared to rural women (OR = 2.38; 95%CI: 1.74, 3.24). Similarly, women in clusters having BEmOC (health center) within 2 hours distance on foot in average were more likely to use skilled care (OR = 1.61; 95%CI: 1.12, 2.33). However, having CEmOC (hospital) within 2 hours distance on foot in average had non-significant effect on skilled care use (OR = 1.36; 95%CI: 0.74, 2.51).

Among the lower (individual) level variables, maternal education, husband’s occupation, wealth quintiles, gravida, inter-birth interval, knowledge of key danger signs during labor, ANC visit and BP & CR were found to have statistically significant association with skilled care use. Having primary (OR = 1.37; 95%CI: 1.10, 1.71), secondary (OR = 3.48; 95%CI: 2.22, 5.45) or tertiary (OR = 3.97; 95%CI: 1.38, 11.46) educations were found to increase the likelihood of skilled care use significantly as compared to not having formal education. Similarly, women whose husbands were employed (OR = 3.26; 95%CI: 2.15, 4.94) or merchants (OR = 2.27; 95%CI: 1.60, 3.23), were more likely to use skilled care as compared to those whose husbands were farmers. Women in the 3rd wealth quintiles (OR = 1.73; 95%CI: 1.12, 1.84) and 4th quintiles (OR = 1.28; 95%CI: 1.11, 1.73) were more likely to use skilled care as compared to those in the lowest quintile.

Gravida and inter-birth interval were among the individual level obstetric factors found to have significant association with skilled care use. Mothers with experience of 1-4 pregnancies (OR = 0.41; 95%CI: 0.26, 0.66) and 5 or more (OR = 0.45; 95%CI: 0.26, 0.76) were less likely to use skilled care as compared to primi-gravida mothers. Inter-birth interval of >48 months was found to increase the likelihood of skilled care use as compared to interval of <24 months (OR = 2.18; 95%CI: 1.30, 3.63).

Knowledge of key danger signs during labor was the other determinant of skilled care use. Women who knew 3 or more key danger signs during labor were more likely to use skilled care as compared to those who didn’t know any key danger signs (OR = 1.59; o5%CI: 1.05, 2.43). Similarly, having 1-3 ANC visits (OR = 1.63; 95%CI: 1.23, 2.18) and ≥4 visits (OR = 3.10; 95%CI: 2.31, 4.16) during pregnancy were found to increase the likelihood of skilled care use significantly. After controlling all the necessary variables in the mixed-effects multilevel model, birth preparedness and complication readiness plan had significant effect on skilled care use. Women who were well-prepared during pregnancy were more likely to use skilled care as compared to those who were not well-prepared (OR = 1.32; 95%CI: 1.03, 1.68) (Table 4).

**Table 4:** Multilevel analysis of factors associated with skilled care use among respondents, Jimma Zone, Southwest Ethiopia, September 2012-April 2013 (n = 3472)

**Factors**	**Skilled care use**			**Crude OR(95%CI)**	**Adjusted OR(95%CI)**
	**Skilled care (n = 1064) n(%)**	**Non skilled care (n = 2408) n(%)**	**Total (n = 3472) n(%)**		
Leve-2 (communal) variables					
Place of residence					
Rural	431 (16.5)	2187 (83.5)	2618 (100.0)	1.00	1.00
Urban	633 (74.1)	221 (25.9)	854 (100.0)	14.49 (12.05, 17.54)	2.38 (1.74, 3.24)
Distance from Health center (on foot)					
≤ 2 hours	917 (37.5)	1531 (62.5)	2448 (100.0)	3.57 (2.95, 4.33)	1.61 (1.12, 2.33)
>2 hours	147 (14.4)	877 (85.6)	1024 (100.0)	1.00	1.00
Distance from Hospital (on foot)					
≤ 2 hours	356 (73.7)	127 (26.3)	483 (100.0)	9.03 (7.25, 11.25)	1.36 (0.74, 2.51)
>2 hours	708 (23.7)	2281 (76.3)	2989 (100.0)	1.00	1.00
Leve-1 (individual level) variables-socio-demographic & economic					
Age (in years)					
<20	77 (44.3)	97 (55.7)	174 (100.0)	1.00	1.00
20-29	740 (33.2)	1487 (66.8)	2227 (100.0)	0.63 (0.46, 0.86)	1.32 (0.85, 2.07)
≥30	247 (23.1)	824 (76.9)	1071 (100.0)	0.38 (0.27, 0.53)	1.34 (0.80, 2.23)
Ethnicity					
Oromo	775 (25.5)	2266 (74.5)	3041 (100.0)	1.00	1.00
Others	289 (67.1)	142 (32.9)	431 (100.0)	5.95 (4.79, 7.39)	1.21 (0.80, 1.84)
Religion					
Muslim	754 (24.9)	2278 (75.1)	3032 (100.0)	1.00	1.00
Others	310 (70.5)	130 (29.5)	440 (100.0)	7.20 (5.78, 8.98)	1.33 (0.88, 2.04)
Educational status					
No Formal Education	341 (18.1)	1538 (81.9)	1879 (100.0)	1.00	1.00
Primary (1-8)	454 (35.7)	818 (64.3)	1272 (100.0)	2.50 (2.12, 2.95)	1.37 (1.10, 1.71)
Secondary (9-12)	208 (81.6)	47 (18.4)	255 (100.0)	19.96 (14.24, 27.98)	3.48 (2.22, 5.45)
Tertiary (>12)	61 (92.4)	5 (7.6)	66 (100.0)	55.03 (24.94,137.97)	3.97 (1.38, 11.46)
Occupation					
House wife	928 (28.2)	2361 (71.8)	3289 (100.0)	1.00	1.00
Employed	72 (88.9)	9 (11.1)	81 (100.0)	20.35 (10.14, 40.87)	0.84 (0.30, 2.31)
Others	64 (62.7)	38 (37.3)	102 (100.0)	4.28 (2.85, 6.45)	0.83 (0.48, 1.44)
Occupation of Husband					
Farmer	371 (15.0)	2102 (85.0)	2473 (100.0)	1.00	1.00
Employed	303 (81.2)	70 (18.8)	373 (100.0)	24.39 (18.52, 32.26)	3.26 (2.15, 4.94)
Merchant	267 (64.8)	145 (35.2)	412 (100.0)	10.42 (8.26, 13.16)	2.27 (1.60, 3.23)
Others	123 (57.5)	91 (42.5)	214 (100.0)	7.63 (5.71, 10.31)	1.50 (0.96, 2.32)
Wealth Index					
1st Quintile (lowest)	148 (21.8)	532 (78.2)	680 (100.0)	1.00	1.00
2nd Quintile	233 (33.0)	472 (67.0)	705 (100.0)	1.77 (1.39, 2.56)	1.20 (0.88, 1.64)
3rd Quintile	222 (32.0)	472 (68.0)	694 (100.0)	1.69 (1.32, 2.16)	1.73 (1.12, 1.84)
4th Quintile	217 (31.2)	478 (68.8)	695 (100.0)	1.63 (1.28, 2.08)	1.28 (1.11, 1.73)
5th Quintile (highest)	244 (35.0)	454 (65.0)	698 (100.0)	1.93 (1.52, 2.46)	1.09 (0.80, 1.48)
Level-1 (Individual)-obstetric					
Gravida (# of pregnancies)					
Primi (1st)	377 (51.3)	358 (48.7)	735 (100.0)	1.00	1.00
2-4	531 (28.9)	1304 (71.1)	1835 (100.0)	0.39 (0.32, 0.46)	0.41 (0.26, 0.66)
>4	156 (17.3)	746 (82.7)	902 (100.0)	0.20 (0.16, 0.25)	0.45 (0.26, 0.76)
Inter-birth interval					
<24 Months	43 (17.1)	209 (82.9)	252 (100.0)	1.00	1.00
24-48 Months	489 (22.5)	1680 (77.5)	2169 (100.0)	1.42 (1.01, 1.99)	1.27 (0.83, 1.95)
>48 Months	155 (49.1)	161 (50.9)	316 (100.0)	4.68 (3.15, 6.95)	2.18 (1.30, 3.63)
Primi gravid	377 (51.3)	358 (48.7)	735 (100.0)	N/A	N/A
Knowledge of key danger signs during labor & delivery					
Not know at least 1 key danger sign	330 (24.2)	1031 (75.8)	1361 (100.0)	1.00	1.00
Know 1-2 key danger signs	607 (33.7)	1192 (66.3)	1799 (100.0)	1.59 (1.36, 1.86)	1.09 (0.86, 1.39)
Know 3-4 key danger signs	127 (40.7)	185 (59.3)	312 (100.0)	2.15 (1.66, 2.78)	1.59 (1.05, 2.43)
Knowledge of key danger signs of neonates					
Not know at least 1 key danger sign	414 (25.8)	1189 (74.2)	1603 (100.0)	1.00	1.00
Knows 1-2 key danger signs	570 (33.9)	1111 (66.1)	1681 (100.0)	1.47 (1.27, 1.71)	1.07 (0.85, 1.35)
Knows 3-4 key danger signs	80 (42.6)	108 (57.4)	188 (100.0)	2.13 (1.56, 2.90)	1.19 (0.71, 1.98)
No. of ANC Visit					
Not at all	110 (13.1)	728 (86.9)	838 (100.0)	1.00	1.00
1-3 times	321 (22.8)	1085 (77.2)	1406 (100.0)	1.96 (1.55, 2.48)	1.63 (1.23, 2.18)
≥4 times	633 (51.5)	595 (48.5)	1228 (100.0)	7.04 (5.60, 8.86)	3.10 (2.31, 4.16)
BP & CR practice					
Not well prepared	425 (18.7)	1844 (81.3)	2269 (100.0)	1.00	1.00
Well prepared	639 (53.1)	564 (46.9)	1203 (100.0)	4.92 (4.21, 5.74)	1.32 (1.03, 1.68)

*N/A* Not Applicable.

## Discussions

The World Health Organization (WHO) strongly recommends that every pregnancy should be delivered under skilled care [16]. The UN’s MDG also had set a target of 90% coverage of skilled care by the end of 2015. But, in this study, the status of skilled care use was found to be very low (17.5%) as compared to this target. This is almost comparable with the findings of other previous studies in the country in which the coverage of skilled care uses were less than 15% [8,17-20]. However, this finding is lower than the findings of similar studies conducted in other developing African countries in which skilled care use ranged from nearly 40-50% [21-24]. The difference may be due to variations in interventions from country to country. This clearly indicated that a lot needs to be done in the study area as well as in the country to increase the status of skilled care use so as to attain the desired target.

Though intention didn’t 100% imply action in skilled care use, delivery planning ahead by identifying place of delivery significantly increased the actual use in this study. The major reason for those who didn’t actually translate their intention in to action was that complication during labor was not predictable. Some of those who planned to give birth at home delivered at health facility because of the occurrence of problems and some of those who planned to go to health facility gave birth at home as their labor was short and didn’t encounter any problem. This finding is consistent with other prior studies where occurrence of problem during labor is the primary reason for women in developing countries to seek skilled care at birth [20,21].

In this study, both the higher level and lower level variables were found to be important determinants of skilled care use suggesting the importance of interventions both at the community as well as individual levels. Among the higher level variables, place of residence and access to BEmOC were the important determinants of skilled care use. This is consistent with other studies conducted before [17-21]. This may be explained by the fact that women in urban areas have more access to skilled care, access to education and media that might have increased their level of risk perception and thereby increased skilled care use.

Among the individual level variables related to socio-demographic and economic characteristics, maternal education, husband’s occupation and wealth quintiles were identified as determinants of skilled care use in this study. These findings are again consistent with other prior studies in the country as well as aboard [17-28]. This may be because, better education increases access to information, risk perception, employment and income which in turn increase health seeking behavior for obstetric complications.

Among the obstetric factors, number of pregnancies (gravida) and inter-birth interval were found to be important determinants of skilled care use in this study. Number of pregnancies had inverse relationship with the rate of skilled care use. Multi-gravida women were less likely to use skilled care. This is consistent with other studies conducted before [17-20]. This may be due to the existence of less risk and less occurrence of prolonged labor as compared to primi-gravida mothers. On the other hand, inter-birth interval of more than 4 years had significant effect in increasing skilled care use. Many international evidences has also suggested that inter-birth interval of 2 years and above significantly improves maternal and child health including skilled care use [21]. This may be due to the reason that mothers may get free time and rest and prepare themselves when they have enough time between pregnancies and utilize the services.

Knowledge of key danger signs during labor, ANC visit and BP & CR plan were also among the individual level variables found to have statistically significant effect on skilled care use in this study. This is also similar with the findings of other previous studies [17-22,29-31]. This can be explained by the fact that women might have received adequate information about danger signs, birth planning and importance of skilled care during ANC visits that facilitated the actual practice at the time of labor. With this, this study revealed that birth preparedness is effective in enhancing skilled care use during delivery if appropriately implemented.

For policy and program implications, this study came up with the evidence that intention implies action in skilled care use; but sometimes this may not be the case as complication during labor is unpredictable. This pointed out to the importance of raising the knowledge of women on key danger signs, risk perception and enhancing birth planning by every pregnant women and her family so that every delivery will be conducted under skilled care.

This study has its own strengths in that the design was prospective follow-up study that measured fresh memory of the mothers and minimized recall bias. It also used large sample size that resulted in high power for the multilevel analysis. In addition, strong statistical model (mixed-effects multilevel analysis) was used to handle clustering effects. This study may have its own limitations in that it measured access to health facility, but didn’t address the quality of the health facility which could have been one of the determinant factors, which may be area for further research. In addition, access to CEmOC had non-significant effect on skilled care use. This might be because, the distance was measured in women’s oral approximate report and no instruments like GPS were used to know the exact distance. There might also be some unobserved cluster (community) level factors like road and transport availability that might have affected this relation which needs to be considered in future researches.

## Conclusions

This study revealed that the status of skilled care use (17.5%) in the study area is still very low as compared to MDG target. The study also revealed that both community level and individual level factors were important determinants of skilled care use. The study also found that birth preparedness plan has significant effect in enhancing skilled care use during delivery. Place of residence, access to BEmOC, maternal education, husband’s occupation, wealth quintiles, number of pregnancy, inter-birth interval, knowledge of key danger signs during labor and ANC use were identified as factors affecting skilled care use. IEC/BCC to increase ANC use, enhancing BP & CR plan with particular emphasis to key danger signs and improving family planning use for birth spacing are recommended as short term interventions. As a long term interventions, female education and improving access to BEmOC with midwifery skills are recommended.

## Abbreviations

ANC: Antenatal Care; BP: Birth Preparedness; CR: Complication Readiness; HEW: Health Extension Worker; OR: Odds Ratio; COR: Crude Odds Ratio; SVD: Spontaneous Vaginal Delivery.

## Competing interests

The authors declare that they do not have any competing interest concerning the findings of the study.

## Authors’ contributions

GTD involved in the conception of the study. GTD, MFA and AWY involved in the design, data collection process, analysis and interpretations of the findings. GTD prepared the initial manuscript which latter be read and edited by MFA and AWY. The final manuscript was read and approved by all authors.
